# Inherently high uncertainty in predicting the time evolution of epidemics

**DOI:** 10.4178/epih.e2021014

**Published:** 2021-02-08

**Authors:** Seung-Nam Park, Hyong-Ha Kim, Kyoung Beom Lee

**Affiliations:** 1 Korea Research Institute of Standards and Science, Daejeon, Korea; 2 University of Science and Technology, Daejeon, Korea

**Keywords:** COVID-19, Epidemiology, Mathematical model, Uncertainty

## Abstract

**OBJECTIVES:**

Amid the spread of coronavirus disease 2019 (COVID-19), with its high infectivity, we have relied on mathematical models to predict the temporal evolution of the disease. This paper aims to show that, due to active behavioral changes of individuals and the inherent nature of infectious diseases, it is complicated and challenging to predict the temporal evolution of epidemics.

**METHODS:**

A modified susceptible-exposed-infectious-hospitalized-removed (SEIHR) compartment model with a discrete feedback-controlled transmission rate was proposed to incorporate individuals’ behavioral changes into the model. To figure out relative uncertainties in the infection peak time and the fraction of the infected population at the peak, a deterministic method and 2 stochastic methods were applied.

**RESULTS:**

A relatively small behavioral change of individuals with a feedback constant of 0.02 in the modified SEIHR model resulted in a peak time delay of up to 50% using the deterministic method. Incorporating stochastic methods into the modified model with a feedback constant of 0.04 suggested that the relative random uncertainty of the maximum fraction of infections and that of the peak time for a population of 1 million reached 29% and 9%, respectively. Even without feedback, the relative uncertainty of the peak time increased by up to 20% for a population of 100,000.

**CONCLUSIONS:**

It is shown that uncertainty originates from stochastic properties of infections. Without a proper selection of the evolution scenario, active behavioral changes of individuals could serve as an additional source of uncertainty.

## INTRODUCTION

Everyday life has changed dramatically since the coronavirus disease 2019 (COVID-19) outbreak, especially after it was declared as a pandemic by the World Health Organization [1,2]. The disease has crucially affected the world economy and human interactions more broadly. The highly infectious nature of this novel virus at its initial stages caused both the general public and health authorities to panic.

The stronger the fear triggered by a disease, the greater the concern as to when the disease will be eradicated. With the hope of providing a scientifically-based answer to this question for COVID-19, several rapidly published reports attempted to estimate the epidemic properties and temporal evolution of the disease based on mathematical models [3-5]. Policy-makers were able to take action to mitigate the impact of the disease thanks to these efforts made by scientists. Although reasonable assumptions and logical models have been applied to predictions of the temporal evolution of COVID-19, slight discrepancies between the predictions and the reality may undermine scientists’ efforts. These discrepancies could be caused partially by imperfections of the models and mainly because of the intrinsic complexity of the relationship between the disease and the responses of individuals and quarantine authorities.

Deterministic and stochastic methods have been used to model infectious diseases. The deterministic method is based on a system of ordinary differential equations (ODEs). The time evolution of its dependent variables is inevitably determined by a set of initial values. Deterministic models treat variables (e.g., the size of the population in a state or a compartment) as continuous variables. Stochastic methods consider noise in the observations and processes of infectious diseases, where the time evolution of dependent variables can be treated as continuous or discrete depending on the model [6]. However, despite the scattering present in their results, stochastic methods are advantageous when attempting to gain insights into the prediction uncertainties of disease progress in reality with a limited population size. Although the results of stochastic methods asymptotically converge to those of deterministic methods as the population size increases, stochastic methods can uniquely demonstrate extinction of the disease at the final stage [6].

Both deterministic and stochastic models are dynamic systems, the stability of which is vulnerable to variations in model parameters. In particular, it should be noted that the transmission rate in the model is sensitive to behavioral changes of the susceptible hosts due to the fear of infections. Several studies have incorporated behavioral changes of individuals into their models [7-9]. In addition, for simplicity, models assume homogeneous mixing of the individuals in a community. In order to account for the fact that mixing is heterogeneous in reality, however, there is a need for more complex models, such as a matrix form of the transmission rate or an introduction of an effective size of the population to the simple model. A simple treatment of mixing with a single value of the transmission rate results in another source of uncertainty in forecasting the progress of a pandemic [10]. Uncertainties in other model parameters are additional sources of overall uncertainty of the predictions.

In this paper, we point out the inherently high uncertainty in predicting the temporal evolution of infectious diseases. First, we propose a simple mathematical model that can take the behavioral change of individuals into account. How behavioral changes induce delays in the epidemic peak is shown for both deterministic solutions and stochastic evolution. Second, the randomness of the spread is highlighted by considering the statistics of the stochastic results. In addition, we present a comparison of two algorithms that have widely been applied to the stochastic evolution of discrete-state Markov chains.

## MATERIALS AND METHODS

### Modified susceptible-exposed-infectious-hospitalized-removed model with a discrete feedback-controlled transmission rate

As a member of the family of compartment models, the susceptible-exposed-infectious-hospitalized-removed (SEIHR) model without demographic characteristics has been applied in studies of COVID-19, wherein individuals are partitioned into the following compartments: susceptible *S*(*t*), exposed *E*(*t*), infected *I*(*t*), hospitalized *H*(*t*), and recovered *R*(*t*) [4,11]. The model was proposed to study childhood diseases under the name of the SEIQR model by Geberry et al. [12] and applied using a stochastic method by Ferrante et al. [13].

We propose a modified SEIHR (M-SEIHR) model that divides the timeline into discrete intervals to account for the effects of individuals’ behavioral changes on the next period, as follows:

(1) $$\left[\begin{array}{c} \dot{S}(t)\\ \dot{E}(t)\\ \dot{I}(t)\\ \dot{H}(t)\\ \dot{R}(t) \end{array}\right]\;=\;\left[\begin{array}{c} -\beta_{j}IS/N\\ \beta_{j}IS/N\;-\;\kappa E\\ \kappa E\;-\;\alpha I\\ \alpha I\;-\;\gamma H\\ \gamma H \end{array}\right]\;,\;t_{j}\;<\;t\;<\;t_{j+1}\;for\;j\;=\;0,\;1,\;2,\;...,\;$$

where *t_j_* refers to the starting point of the period *j* and *S_j_*(*t*) the number of susceptible individuals at time *t* between the period *j* and *j*+1. The parameters *β* and 1/*κ* indicate the infection transmission rate and the mean incubation period, respectively. The isolation rate *α* is mostly determined by the transition from the infected to the hospitalized, and partially by the transitions to death and to the recovered without hospitalization. A higher value of the isolation rate decreases the size of the infected population, whereas a higher *β* value increases the size of the infected population. The ratio of these parameters is defined as the reproduction number ratio, *R*_0_=*β*/*α*, which allows us to roughly forecast the progress of the disease at the initial stage. Assuming that a single individual has been exposed among a population size of *N*, the initial condition of equation (1) is set as

(2) [S(0), E(0), I(0), H(0), R(0)]= [N−1, 1, 0, 0, 0],

to meet the constraint of

(3) S(t)+E(t)+I(t)+H(t)+R(t)=N.

As shown in equation (1), the M-SEIHR model assumes that the temporal evolution of the number of each compartment during each period is governed by the same form of ODE as the original SEIHR model. Starting from the initial condition of equation (2), the initial condition of the ODE during each period is given by its solution at the endpoint of the previous period.

The M-SEIHR model further assumes that the transmission rate at each period is adjusted at the starting point of the period. To simplify the calculations to achieve the purposes of this study, the adjustment is assumed to be proportional to the relative increase rate of the number of the infected multiplied by *δ*, as follows:

(4) $$\beta_{j}\;=\;\beta_{j-1}\;\left\{1\;-\;\delta \frac{I(t_{j})-I(t_{j-1})}{I(t_{j})}\right\},\;for\;j\;=\;1,\;2,\;3,\;...$$

During a period, the susceptible are expected to be exposed to news media and then to change their contact behavior due to their fear of infection, which eventually leads to an adjustment of the transmission rate. The adjusted transmission rate is continuously fed back into equation (1) to calculate the evolution for the next period until the last period is reached. This recursion resembles the proportional control of dynamic systems. Therefore, the *δ* is called the feedback constant hereafter. If *δ*=0, the M-SEIHR model reduces to the original ODEs. The temporal evolution described by equation (1) during each period can be calculated by a deterministic method relying on the ODEs or one of the stochastic methods explained in the next section.

In reality, in Korea, because news media daily report the number of newly infected cases, most of whom are hospitalized (or transferred to treatment centers), it is possible to make the M-SEIR model more practical by replacing the number of the infected in equation (4) with the number of the hospitalized. The replacement results in an explicit time dependence of the variables in equation (1) on time (*t_j_* –1), which requires more complicated methods instead of popular numerical methods for the ODE such as the Runge-Kutta fourth-order or the adaptive Runge-Kutta algorithm.

### Discrete-state Markov chain method

This study adopted two approaches to implement the discretestate Markov chain method: an event-driven approach (EDA) and a discrete-time stochastic method (DSM). In the EDA, the transitions between the states or compartments (S, E, I, H, R) are stochastically determined by the transition rates described in the ODEs of equation (1). The events take place following the Poisson distribution, which is reflected in Gillespie’s algorithms such as the first-reaction algorithm and the efficient algorithm [6,14]. We simulated the underlying ODE of equation (1) using the direct algorithm, which is the most classical version and requires the generation of two uniform random numbers at every time step. One of the random numbers determines which transition happens and the other when the transition takes place. The algorithm has been well summarized by Keeling & Rohani [6].

The DSM has been applied to study a behavior-disease model by Perra et al. [7] and recently for COVID-19 by He et al. [11]. Referring to both of those studies, the DSM was used by assuming a simple binomial distribution of the transition of individuals in discrete units of time to produce a set of chains through the progress of the infection. Each member in the compartment at time *t_j_* has a probability of the transition rate multiplied by the time interval ∆*t*=*t*_*j*+1_−*t_j_* during the time interval between *t_j_* and *t*_*j*+1_ and then jumps to the next compartment at time *t_j_*. For example, the number of individuals jumping from the susceptible to the exposed compartment during the interval ∆*t* is generated following the binomial distribution *Bin* [*S, β∆tI*(*t_j_*)/*N*], which forces a decrease in the number of susceptible individuals and an increase in the number of exposed individuals. Similarly, considering all possible transition rates, a Markov chain with the net numbers in the compartments [*S*(*t*), *E*(*t*), *I*(*t*), *H*(*t*), *R*(*t*): *t*=0, *t*_1_, *t*_2_, …] is formed. The next state of the system is determined by the current state through a part of the Markov chain relations. The difference equations connecting the chains are described up to the number of the infected for our purposes as:

(5) $$S\;(t_{j+1})=S(t_{j})-Bin\;[S(t_{j}),\;\beta ∆tI(t_{j})/N],\;$$

(6) $$E\;(t_{j+1})=E(t_{j})+Bin\;[S(t_{j\;}),\;\beta ∆tI{(}_{j}t)/N]-Bin\;[E(t_{j}),\;\kappa ∆t]$$

(7) $$I\;(t_{j+1})=I(t_{j})+Bin\;[E(t_{j}),\;\kappa ∆t]-Bin\;[I(t_{j}),\;\alpha ∆t],$$

which shares the same initial condition and parameters as equation (2). Because ∆*t* can be arbitrarily selected, matching the time intervals in both the DSM and the M-SEIHR model enables the DSM to be easily incorporated into the M-SEIHR model.

### Ethics statement

Requiring the approval of the Institutional Review Board was waived as this study carried out the numerical experiment based on a mathematical model to predict the time evolution of epidemics.

## RESULTS

### Peak time of infections

**Figure 1.**
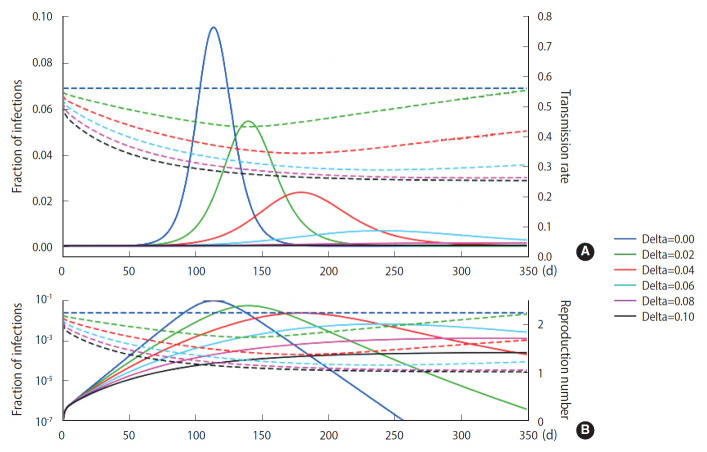
Deterministic temporal evolution of an infectious disease in terms of the fraction of infections (solid lines) for various feedback constants taking into account individuals’ behavioral changes once a day (in B are log-scaled). The dotted lines represent the adjusted transmission rate (A) and the reproduction number (B), respectively.

Using the M-SEIHR model, we investigated the peak time delay of infections using a set of model parameters (*β*=0.56, *κ*=1/4 [1/d], and *α*=1/4 [1/d]) that is known to be suitable for describing the time evolution of COVID-19 [3]. The transmission rate was obtained from the reproduction number ratio in the COVID-19 cases of Daegu and North Gyeongsang Province in Korea in its early stage [4]. The feedback constant *δ* was assumed to change from 0.00 to 0.10, with an interval of 0.02. The solutions for the peak time delay are shown in Figure 1. As the feedback constant *δ* increases, the peak is drastically delayed and the fraction of infections significantly decreases. The dotted line represents the transmission rate, which is adjusted once a day as the relative infection rate changes.

**Figure 2.**
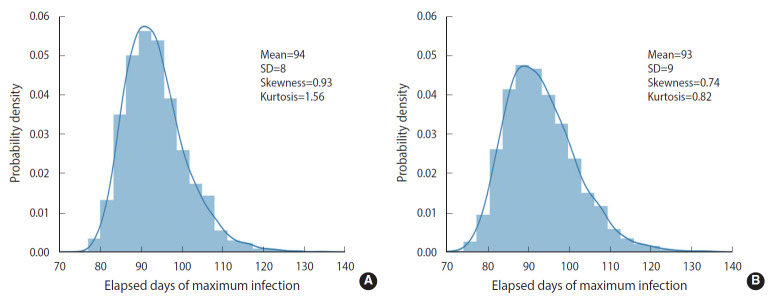
Comparison of two stochastic methods with 5,000 computational trials and without any feedback (*δ*=0.00): the discrete-time stochastic method (A) versus the event-driven approach (B). SD, standard deviation.

To simulate more realistic situations, stochastic methods are essential. However, computational speed limits the choice of methods. To compare the calculation result and speed of the EDA with those of the DSM, we conducted 5,000 computational trials for a population size of 100,000 without any feedback on behavioral changes (*δ*=0). The calculation results are represented in terms of a probability density distribution (PDF) of the elapsed days of maximum infections, as shown in Figure 2. The EDA and DSM took 520 s and 13 s using a laptop computer, respectively. The DSM was 40 times faster than the EDA. Nevertheless, the PDFs showed a discrepancy of the means (1 day) that was insignificant and well within the standard deviation of the DSM (8 day). Therefore, the stochastic simulations were conducted using the DSM.

### Random nature

**Figure 3.**
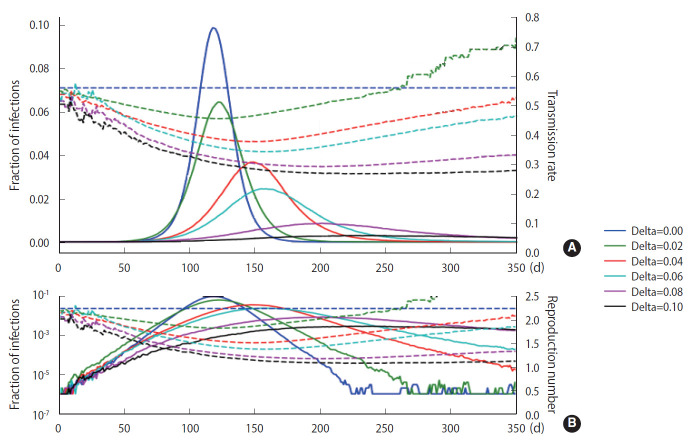
The stochastic model implemented using the same parameters as in Figure 1, which was obtained by the chain binominal method. Its discrepancies with Figure 1 are caused by the random nature of the method (B is the log-scaled of A).

As discussed in Section 2, a stochastic method can be applied in the M-SEIHR model. For a comparison with the deterministic method, we repeated the calculations with the same model parameters as those used in Figure 1 and obtained the peak time delays. Figure 3 represents the peak time delays in a typical set of pandemic curves, which show clear randomness as we repeat the computation. Owing to the randomness of the stochastic methods, the shift of the peaks does not appear as smooth as in Figure 1. Furthermore, the data derived from the maximum infected fraction do not agree with the data from Figure 1. Such discrepancies may be an origin of the uncertainty in predicting the temporal evolution of infectious diseases.

**Figure 4.**
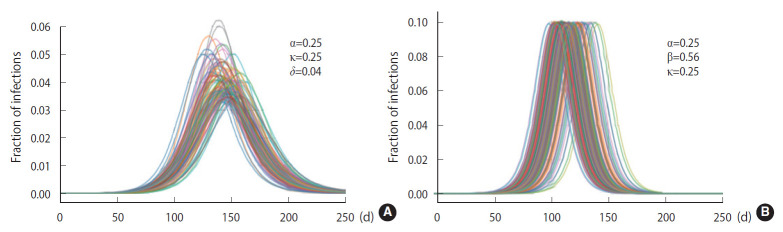
Randomness in the time evolutions of the disease with a feedback control of the transmission rate set as δ=0.04 (A) and without any feedback control (B).

**Figure 5.**
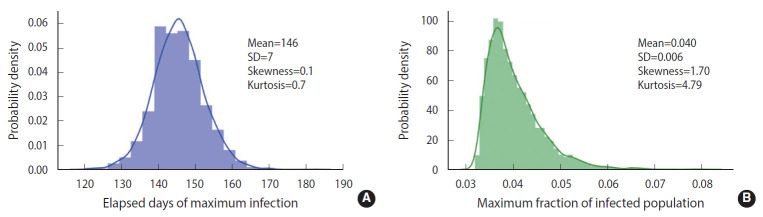
Probability density distributions of the respective elapsed days in reaching the maximum (A) and the maximum fraction of infected populations (B). The feedback with *δ*=0.04 was imposed in the simulation.

To demonstrate the randomness in further detail, repeated calculations of the temporal evolution of the infection were carried out and depicted in Figure 4, which traces 200 evolutions. Figure 4A corresponds to the case of the M-SEIHR model with *δ*=0.04. Both the peak times and the respective maximum fraction on the peak appear with a dispersion. Figure 4B presents the case without any feedback (*δ*=0.00), which does not show scattering in the maximum fraction of infection, but does show significant scattering in the peak times. Figure 4 implies the possibility of having PDFs of the peak times and the maximum fractions of the infected population. Assuming a population of 1,000,000, we repeated the calculations 5,000 times to obtain both PDFs, as shown in Figure 5. This calculation imposed a moderate level of feedback with a feedback constant (*δ*) of 0.04.

**Figure 6.**
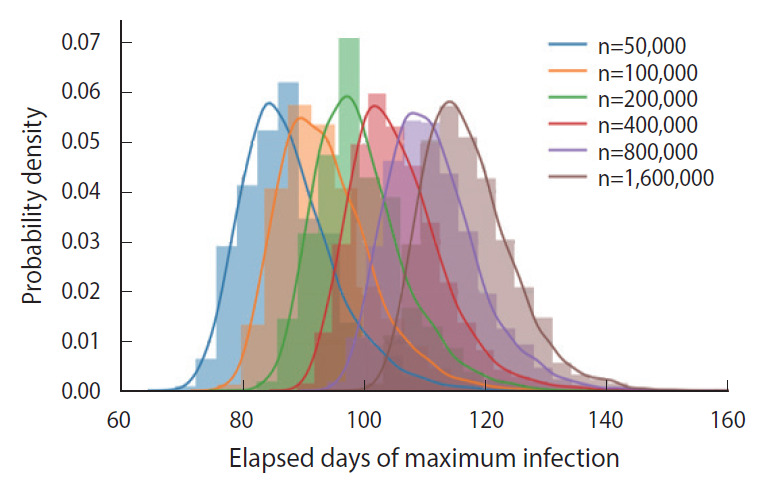
Probability density distribution of the peak time delays of the infections for various sizes of the initial population of the susceptible.

In essence, different susceptible populations result in different peak times of the infection. The effective population size is determined by a degree of the mixing of the infected and the susceptible. We calculated the PDFs of the peak infection time for various sizes of an initial susceptible population ranging from 50,000 to 1,600,000, as shown in Figure 6. Each PDF was obtained from a trial sample of 5,000 calculated by the DSM without feedback (*δ*=0.00).

## DISCUSSION

As shown in Figure 1, we observed delays of the peak time in the progress of an infection and reductions of the maximum fraction of the progress using the M-SEIHR model proposed for this study. A feedback constant of 0.02 delayed the peak time up to 50% and reduced the maximum infected fraction to 60%. This trend became more non-linear as the feedback increased. Similar to the action of electrical amplifiers, feedback from individuals’ behavioral changes seem to amplify the uncertainty in predicting the temporal evolution, even in the deterministic model. However, this non-linearity may be accepted as a unique tool that health authorities can utilize to mitigate the disease until an effective vaccine becomes available.

We compared the results simulated by 2 stochastic methods. The DSM was found to be superior to the EDA in terms of computational speed without sacrificing the accuracy of the calculation results. Furthermore, the DSM can be more easily implemented than the EDA in the discrete feedback-controlled transmission algorithm for the M-SEIHR model.

As shown in Figure 2, without any feedback, the randomness of the results showed a relative uncertainty of 20% (*k*=2) in the peak time of infections for a population size of 100,000. When feedback is imposed on the stochastic method, as shown in Figure 5, the maximum fraction of the infections is additionally subject to randomness. The PDFs appear to be significantly skewed with a skewness of 1.70. While the maximum fraction showed a relative uncertainty of 29% (*k*=2), the relative uncertainty in the peak time of infections is 9% (*k*=2) for a population of 1,000,000, which is much less than 20% in the case without any feedback. It is interesting to see that a dynamic system driven by feedback showed a more significant reduction of the randomness in the temporal evolution than a free-running dynamic system without feedback. The feedback seems to act as a kind of driving force in the dynamic system. This is analogous to the fact that the seasonal oscillation of influenza epidemics is maintained for a long period even by stochastic calculations, despite a large degree of randomness due to the resonance of the dynamic system [15]. In this case, it should be noted that a low level of the seasonal oscillation acted as the driving force.

As we can intuitively expect, the peak time of maximum infections is delayed as the size of an initial susceptible population increases, which was calculated as shown in Figure 6. Because a perfectly homogeneous mixing of the susceptible and the infected cannot be achieved, it is difficult to fix the effective size of the population [10], and ambiguity in fixing the population size introduces an additional uncertainty in predicting the peak time.

In summary, there are many sources of uncertainty in estimating both the peak time and size of a pandemic. Individuals’ behavioral changes are the most crucial factor, which could be useful information for health authorities. The stochasticity of the infection process is another key factor contributing to uncertainty. Heterogeneous mixing of the susceptible and the infected introduces an additional uncertainty in fixing the effective number of the susceptible. Nevertheless, it should be noted that even if mathematical models cannot avoid errors in forecasting the progress of infectious diseases, they have provided many clues to fighting these diseases.

## References

[1] World Health Organization (2020). WHO Statement regarding cluster of pneumonia cases in Wuhan, China. https://www.who.int/china/news/detail/09-01-2020-who-statement-regarding-cluster-of-pneumonia-cases-in-wuhan-china.

[2] World Health Organization WHO Director-General’s opening remarks at the media briefing on COVID-19 - 11 March 2020. https://www.who.int/director-general/speeches/detail/who-director-general-s-opening-remarks-at-the-media-briefing-on-covid-19---11-march-2020.

[3] Ki M, Task Force for 2019-nCoV (2020). Epidemiologic characteristics of early cases with 2019 novel coronavirus (2019-nCoV) disease in Korea. Epidemiol Health.

[4] Choi S, Ki M (2020). Estimating the reproductive number and the outbreak size of COVID-19 in Korea. Epidemiol Health.

[5] Kim S, Seo YB, Jung E (2020). Prediction of COVID-19 transmission dynamics using a mathematical model considering behavior changes in Korea. Epidemiol Health.

[6] Keeling M, Rohani P (2008). Modeling infectious diseases in humans and animals.

[7] Perra N, Balcan D, Gonçalves B, Vespignani A (2011). Towards a characterization of behavior-disease models. PLoS One.

[8] Poletti P, Ajelli M, Merler S (2011). The effect of risk perception on the 2009 H1N1 pandemic influenza dynamics. PLoS One.

[9] Fenichel EP, Castillo-Chavez C, Ceddia MG, Chowell G, Parra PA, Hickling GJ (2011). Adaptive human behavior in epidemiological models. Proc Natl Acad Sci U S A.

[10] Cui J, Zhang Y, Feng Z (2019). Influence of non-homogeneous mixing on final epidemic size in a meta-population model. J Biol Dyn.

[11] He S, Tang SY, Rong L (2020). A discrete stochastic model of the COVID-19 outbreak: forecast and control. Math Biosci Eng.

[12] Gerberry DJ, Milner FA (2009). An SEIQR model for childhood diseases. J Math Biol.

[13] Ferrante M, Ferraris E, Rovira C (2016). On a stochastic epidemic SEIHR model and its diffusion approximation. TEST.

[14] Gillespie DT (1976). A general method for numerically simulating the stochastic time evolution of coupled chemical reactions. J Comput Phys.

[15] Dushoff J, Plotkin JB, Levin SA, Earn DJ (2004). Dynamical resonance can account for seasonality of influenza epidemics. Proc Natl Acad Sci U S A.

